# RT-QuIC detection of CWD prion seeding activity in white-tailed deer muscle tissues

**DOI:** 10.1038/s41598-021-96127-8

**Published:** 2021-08-18

**Authors:** Manci Li, Marc D. Schwabenlander, Gage R. Rowden, Jeremy M. Schefers, Christopher S. Jennelle, Michelle Carstensen, Davis Seelig, Peter A. Larsen

**Affiliations:** 1 Department of Veterinary and Biomedical Sciences, University of Minnesota, 1971 Commonwealth Ave, Saint Paul, MN 55108 USA; 2 Minnesota Center for Prion Research and Outreach, College of Veterinary Medicine, University of Minnesota, Saint Paul, MN 55108 USA; 3 Veterinary Diagnostic Laboratory, Veterinary Population Medicine Department, University of Minnesota, Saint Paul, MN 55108 USA; 4 Minnesota Department of Natural Resources, 5463 West Broadway, Forest Lake, MN 55025 USA; 5 Department of Veterinary Clinical Sciences, University of Minnesota, Saint Paul, MN 55108 USA

**Keywords:** Biological techniques, Molecular biology, Diseases

## Abstract

Chronic wasting disease (CWD) is a prion disease circulating in wild and farmed cervid populations throughout North America (United States and Canada), Europe (Finland, Norway, Sweden), and South Korea. CWD is a long-term threat to all cervid populations and to cervid hunting heritage, with the potential to cause substantial economic losses across multiple sectors. In North America, hunting and farming industries focused on the processing and consumption of white-tailed deer (WTD) venison are particularly vulnerable to CWD prion contamination, as millions of WTD are consumed annually. Real-time quaking-induced conversion (RT-QuIC) is a highly sensitive assay amplifying misfolded CWD prions in vitro and has facilitated CWD prion detection in a variety of tissues and excreta. To date, no study has comprehensively examined CWD prion content across bulk skeletal muscle tissues harvested from individual CWD infected WTD. Here, we use RT-QuIC to characterize prion-seeding activity in a variety of skeletal muscles from both wild and farmed CWD-positive WTD. We successfully detected CWD prions in muscles commonly used for consumption (e.g., backstrap, tenderloin, etc.) as well as within tongue and neck samples of WTD. Our results suggest that CWD prions are distributed across the skeletal muscles of infected WTD. We posit that RT-QuIC will be a useful tool for monitoring CWD prions in venison and that the method (with additional protocol optimization and high-throughput functionality) could be used to reduce and/or prevent CWD prions from entering animal and human food chains.

## Introduction

Chronic wasting disease (CWD) is an infectious and fatal prion disease transmitted among cervids, including white-tailed deer (WTD; *Odocoileus virginianus*), mule deer, elk, red deer, caribou, reindeer, and moose. The disease is a direct threat to a number of cervid-related multibillion-dollar economic sectors, including both agricultural and hunting industries, and it is now prevalent in the USA and Canada with additional cases in Korea, and Scandinavian regions^1^. As with other transmissible spongiform encephalopathies^2,3^, CWD prion seeds (PrP^CWD^) consist of misfolded cellular prion protein (PrP^C^) which form β-sheet-rich amyloid fibrils through inducing conformational change and polymerization of native PrP^C^. The central nervous system (CNS) typically contains the highest load of prions in a terminally diseased animal in comparison to peripheral tissues and body excreta due to the abundance of PrP^C^ in nervous tissues^4^.

Recent studies have shown that there are compelling reasons to suggest that CWD poses a non-zero risk to a variety of mammals, including humans^1,5^. Challenge experiments using CWD prions have shown that CWD can cause neurodegenerative disease in numerous species, including ferrets, mink, domestic cats, sheep, goats, cows, pigs, and squirrel monkeys^6^. In vitro experiments showed that CWD prions can convert human prion proteins into a misfolded and potentially disease-causing form^5^. For these reasons, as of 2020, both the Food and Drug Administration (FDA) and Food Safety and Inspection Service, United States Department of Agriculture (FSIS, USDA) consider venison from CWD-positive animals as adulterated and unsuitable for consumption^7,8^. While there is no evidence of CWD transmission to humans^1^, the National Institutes of Health and Centers for Disease Control and Prevention suggests that people should not consume known CWD-infected venison.

Currently, CWD diagnosis relies on the identification of Proteinase K (PK)-resistant PrP^CWD^ by enzyme-linked immunosorbent assay (ELISA) and immunohistochemistry (IHC)^9^. These standardized methods for detecting CWD are designed to have consistent protocols with quantified estimates of test accuracy that are scalable to meet the needs of agencies conducting surveillance and monitoring to manage the disease. However, there are limitations to the existing antibody-based diagnostic approaches, namely relatively poor sensitivity as well as the inability to screen biofluids and environmental samples. In the past two decades, the detection of prion seeding activity has been greatly enhanced by highly sensitive methods involving amplification of protein misfolding in vitro, such as protein misfolding cyclic amplification (PMCA) and real-time quaking-induced conversion (RT-QuIC)^9,10^. PMCA uses rodent brain homogenates as the substrate to amplify misfolded prions and Western Blotting as the output^9,11,12^. RT-QuIC utilizes recombinant PrP^C^, commonly from rodent sources, as the substrate for prion amyloid formation, the real-time reporting of which is enabled by thioflavin T (ThT) binding and detection^13–15^. Although RT-QuIC demonstrates unparalleled detection sensitivity and specificity for brain and lymphoid tissues, it has lower sensitivity for other sample types; this has in-part been ascribed to lower prion density and RT-QuIC reaction inhibitors^16^. Importantly, the inhibitory effect of certain tissues—likely due to their biochemical compositions^16,17^, such as blood^18–20^ and saliva, seemed to be specific for RT-QuIC but not PMCA^16^. Dilutions (diluting out inhibitory effects) and phosphotungstic acid (PTA) precipitation are commonly used to increase RT-QuIC sensitivity by enriching for prions and overcoming effects of RT-QuIC inhibitors^15–17,20^, although other methods exist^18,21^.

CWD prions have previously been identified in a variety of tissue types and excreta using RT-QuIC, such as the CNS, third eyelids, and feces^22–25^. Prior studies focused on the detection of CWD prions in skeletal muscle using immunodetection methods have produced mixed results^26–29^. PMCA was used to amplify CWD prions in hindlimb muscles from two WTD^30^. Skeletal muscle tissues from CWD-infected deer contain infectious prions as determined in transgenic mice bioassay^29^. Despite clear advantages of RT-QuIC as a screening method^9,31,32^, no comprehensive reports are available for detecting CWD prions using RT-QuIC in skeletal muscle.

Cervid skeletal muscles are consumed by a growing population of hunters and restaurant clientele and have become a common ingredient in pet food (e.g., commercial cat and dog food). At the time of this publication, there are no guidelines regarding venison-based detection of CWD and associated food-product surveillance. This observation, combined with the limitations of existing CWD diagnostic tools (e.g., ELISA and IHC), has resulted in a situation whereby venison processing can occur without the knowledge of an animal’s CWD status, and it is estimated that at least 15,000 CWD positive cervids are consumed in the USA annually^1^. Underscoring this statistic was a well-documented 2005 exposure event in which over 200 participants at a Sportsmen’s feast consumed CWD-positive venison^33^. Current estimates indicate a 20–50% CWD prevalence rate in harvested WTD from focal areas of southern Wisconsin, however, only 1 out of 3 are tested for the disease^33^. Collectively, these observations highlight the need for post-harvest production-level monitoring of cervid products used for human and animal consumption.

Here, we examine the utility of RT-QuIC for the detection of CWD prions within a broad set of WTD skeletal muscle tissues, including those frequently used for both human and animal consumption. We report the RT-QuIC results for muscles sampled from the neck (*brachiocephalicus/sternocephalicus*) of wild WTD with known CWD status. Further, we investigated whether CWD prion deposition is limited to certain groups of muscles or if it is more generalized by using multiple WTD skeletal muscle groups across the body, including muscles from the tongue, forelimb (*suprascapularis*), backstrap (*longissimus dorsi*), tenderloin (*psoas major*), and hindlimb (*semimembranosus/semitendinosus*) from both wild and farmed CWD positive animals independently determined by ELISA and/or IHC.

## Results

### RT-QuIC detection of CWD prions in unilateral skeletal muscles from the neck of wild WTD

**Figure 1:**
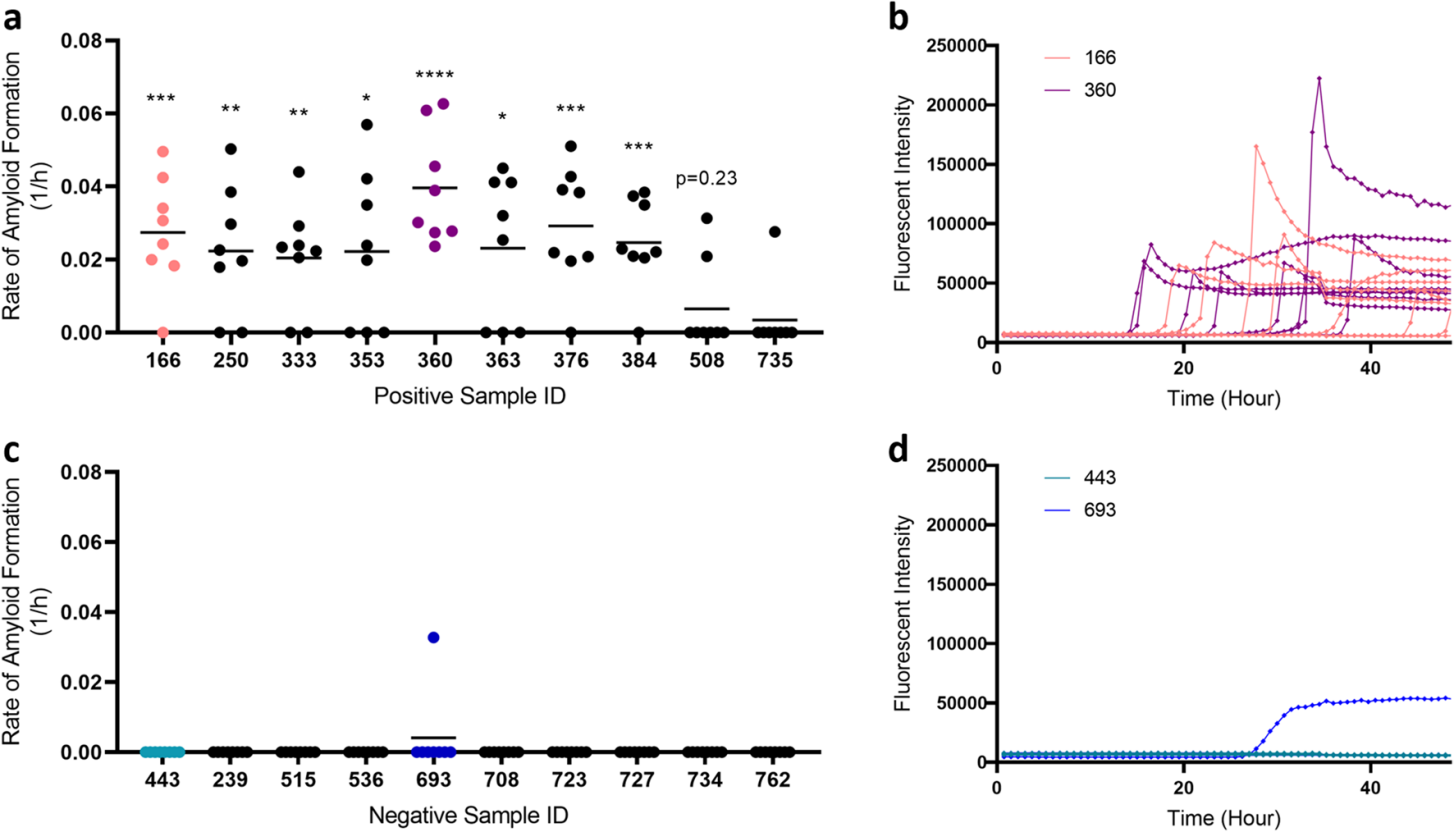
Detection of prion seeding activity in unilateral neck muscles from white-tailed deer. (**a**) Rate of amyloid formation (1/h) was plotted using data collected from CWD-positive animals. Statistical significance was obtained through comparing with rate of amyloid formation with the respective negative controls on the same plate (****p < 0.0001; ***p < 0.001; **p < 0.01; *p < 0.05). (**b**) Examples of real-time fluorescence readings from positive animals (sample IDs 166 and 360). (**c**) Rate of amyloid formation (1/h) from CWD-negative animals. (**d**) Examples of real-time fluorescence readings from negative animals (443 and 693); plotted as described in (**b**) and showing one of eight wells for sample 693 having amyloid seeding activity (not significant).

We first developed a PrP^CWD^ enrichment protocol for muscles—based on previous work^20,25^—herein referred to as the freeze–thaw method as it consists of several rapid freeze–thaw cycles prior to PTA precipitation. To test the performance of the freeze–thaw method, we processed unilateral muscles collected from the neck (*brachiocephalicus/sternocephalicus*) of 10 CWD-positive and 10 CWD-negative wild WTD (Table 1) and analyzed the resultant homogenates using RT-QuIC. We found that we could detect significant prion seeding activity in 8 out of 10 (80%) samples from 10 different CWD-positive animals (Table 1; Fig. 1a) with relatively consistent fluorescent readings (Fig. 1b). In contrast to animals with official CWD-positive test results (i.e., ELISA and IHC), none of the muscle samples from CWD-negative animals showed statistically significant prion seeding activity in RT-QuIC (Fig. 1b), despite one of eight wells crossing the threshold from a single animal (Fig. 1d).

**Table 1:** RT-QuIC results of WTD neck muscles.

MNPRO ID	ELISA/IHC CWD test result	RT-QuIC result	RT-QuIC wells positive
166	+	***	7/8
250	+	**	6/8
333	+	**	6/8
353	+	*	5/8
360	+	****	8/8
363	+	*	5/8
376	+	***	7/8
384	+	***	7/8
508	+	NS	2/8
735	+	NS	1/8
443	–	–	0/8
239	–	–	0/8
515	–	–	0/8
536	–	–	0/8
693	–	NS	1/8
708	–	–	0/8
723	–	–	0/8
727	–	–	0/8
734	–	–	0/8
762	–	–	0/8

All animals were collected through the Minnesota Department of Natural Resources 2019 agency culling operations. All animals’ medial retropharyngeal lymph nodes were tested for CWD through official regulatory means by ELISA, with IHC confirmation on ELISA positives. Mann–Whitney U test: NS, rate of amyloid formation is not 0 but not statistically significant from the corresponding negative controls; –, rate of amyloid formation is 0 in the given time period; ****p < 0.0001; ***p < 0.001; **p < 0.01; *p < 0.05. The freeze–thaw method was used for sample processing and RT-QuIC was performed at 45 °C.

**Figure 2:**
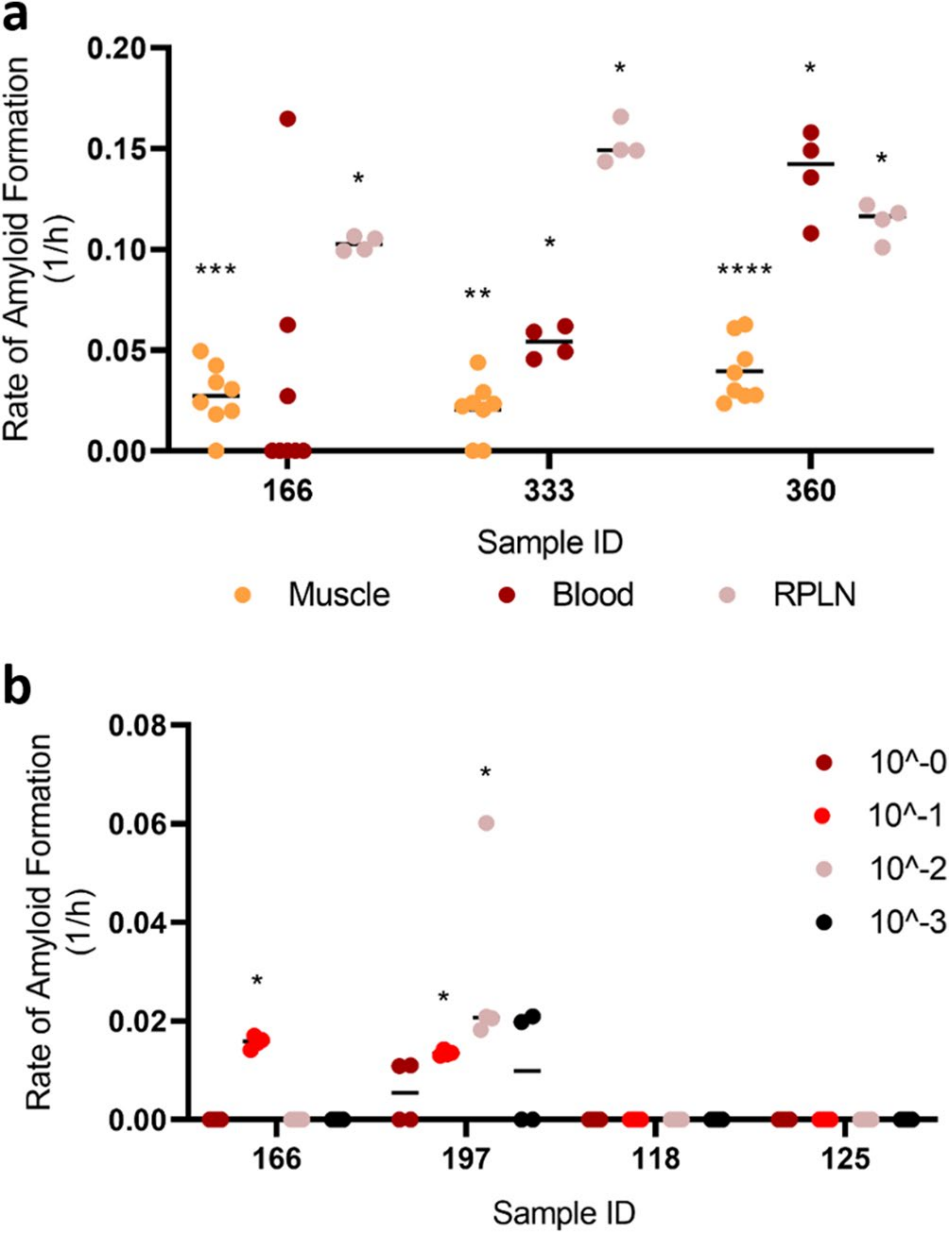
Comparison of prion-seeding activity in RT-QuIC. (**a**) Rate of amyloid formation was compared among neck muscle, blood, and lymphoid tissues of three CWD-positive animals. (**b**) Rate of amyloid formation compared between each sample and the negative control on the same plate. Statistical result for each sample compared with its respective negative control was indicated (****p < 0.0001; ***p < 0.001; **p < 0.01; *p < 0.05). RPLN, medial retropharyngeal lymph nodes.

We then compared the rate of amyloid formation (RAF) among muscles, blood, and lymphoid tissues, all of which were processed using mechanical extraction methods; with methods and results of blood and lymphoid tissues reported by Schwabenlander et al.^32^. We note that although muscles appeared to have a lower RAF, it is possible for an animal to have a statistically positive RT-QuIC result for muscles and lymphoid tissues but not blood (e.g., animal 166; Fig. 2a; see Schwabenlander et al. In press). 1:10 dilution of the enriched homogenates (after NaPTA precipitation) was chosen because of its consistency in producing results in different animals (Fig. 2b). The optimal dilutions (a log10-based dilution that would produce the highest RAF for a given sample) of each animal may differ, with dilution factors ranging from 0 to 3 (Fig. 2b). Because the initial concentration of prion seeds added into RT-QuIC reaction is known to affect detectability and RAF^15^, it is then expected that the method presented here—using suboptimal dilutions for some samples—would underestimate the RAF for consistent detection purposes. Indeed, by comparing the RAF between 1:10 dilution of the enriched homogenates and lymphoid tissues, which exhibit RAF 10 times lower than brain homogenates^17^, we found that indirectly calculated brain/muscle ratio was 100–1000 times lower than previously reported in muscles of mice inoculated with different strains of prions (Supplementary Fig. 1)^34^. Because the tenfold dilution for each sample was likely not optimal, the difference could be attributed to a combination of the presence of RT-QuIC inhibitors in muscle tissues and incomplete extraction (Bosque et al. pulverized muscles under liquid nitrogen^34^) in addition to experimental design and species differences. It is also possible that particular CWD prion seeds within our samples were partially degraded during autolysis (discussed further below).

### CWD prions found in muscles from the tongue, neck, mid-trunk, forelimb, and hindlimb of WTD

**Figure 3:**
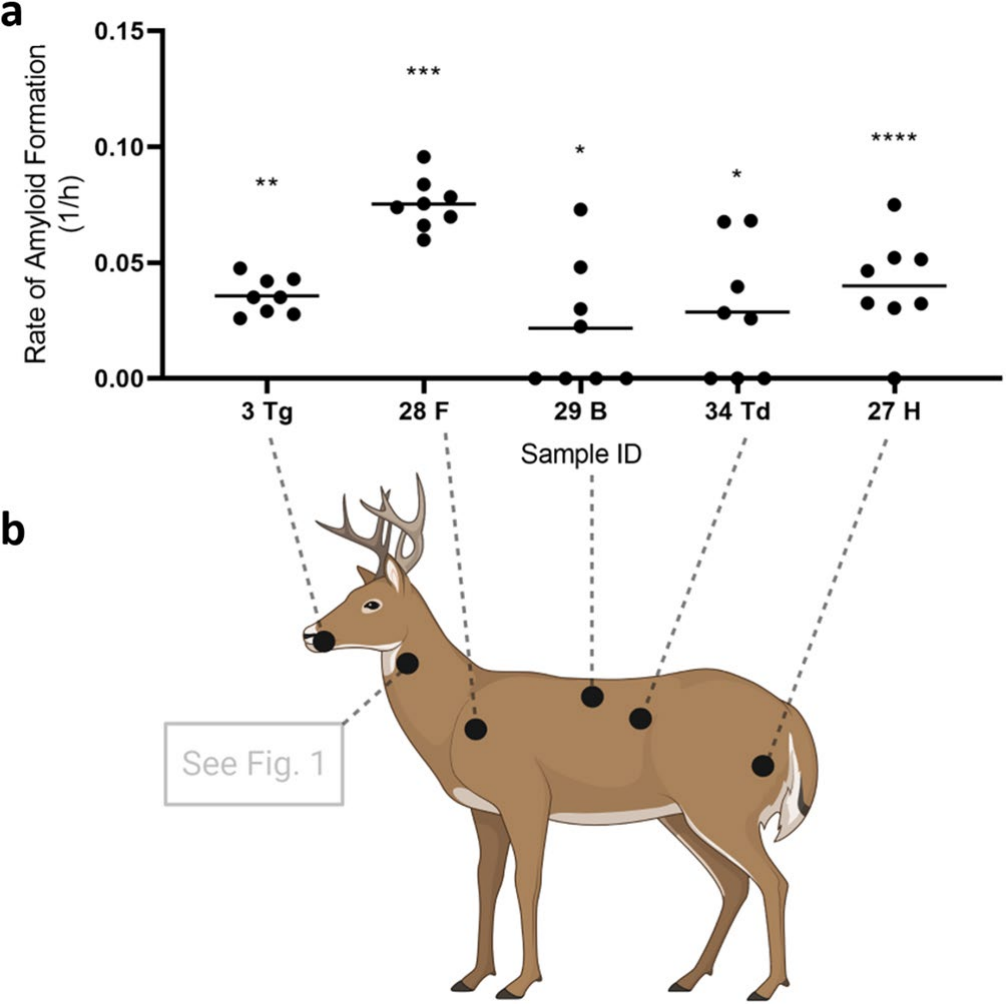
Presence of CWD prions in the muscle tissues of tongue, neck, hindlimb, forelimb, backstrap, and tenderloin. (**a**) Examples of the rate of amyloid formation (RAF) from RT-QuIC positive samples were plotted. Statistical results as compared to the respective negative controls were indicated (****p < 0.0001; ***p < 0.001; **p < 0.01; *p < 0.05). *Tg* Tongue, *F* forelimb, *B* backstrap, *Td* tenderloin, *H* hindlimb. Numbers on X-axis are animal IDs listed in Table 2. (**b**) Prion-seeding activity was detected by RT-QuIC in muscles from the tongue, forelimb, backstrap, tenderloin, and hindlimb. Deer image was created using BioRender.com.

To investigate if the CWD prions are found in WTD skeletal muscles and whether the freeze–thaw method described above can be used to detect prions deposition in other skeletal muscles other than those from the neck, we used a set of muscle tissues from another 10 WTD, including the tongue, forelimb (*suprascapularis*), backstrap (*longissimus dorsi*), tenderloin (*psoas major*), and hindlimb (*semimembranosus/semitendinosus*). In the blinded run, we were able to detect at least one significantly RT-QuIC positive sample in all the muscle groups tested (Table 2; Fig. 3). We observed poor sensitivity of the freeze–thaw method with these particular samples (Table 2; when compared to fresh samples), a result that is likely due to the deteriorated condition of the muscle tissues upon receipt. Nevertheless, we recovered statistically significant RT-QuIC results for a variety of muscle groups and we therefore conclude that PrP^CWD^ occurs broadly throughout the skeletal muscles of infected WTD (Fig. 3b) and are not limited to specific muscle groups, as previously reported in mouse models^34^.

**Table 2:** RT-QuIC results of various WTD muscle groups.

ID	Region	MNPRO ID	ELISA and/or IHC CWD test result	RT-QuIC result	RT-QuIC wells positive
1	F	287	+	–	0/8
2	H	287	+	NS	3/8
3	Tg	287	+	***	8/8
4	B	287	+	–	0/8
5	F	288	+	NS	1/8
6	H	288	+	NS	1/8
7	B	288	+	NS	1/8
8	F	289	+	NS	1/8
9	H	289	+	–	0/8
10	B	289	+	–	0/8
11	F	290	+	–	0/8
12	H	290	+	NS	2/8
13	Tg	290	+	–	0/8
14	Td	290	+	–	0/8
15	H	295	+	NS	1/8
16	F	295	+	–	0/8
17	B	295	+	NS	1/8
18	H	296	+	–	0/8
19	F	296	+	–	0/8
20	B	296	+	–	0/8
21	H	297	+	NS	1/8
22	F	297	+	NS	1/8
23	B	297	+	–	0/8
24	H	298	+	NS	1/8
25	F	298	+	–	0/8
26	B	298	+	–	0/8
27	H	307	+	***	8/8
28	F	307	+	****	8/8
29	B	307	+	*	4/8
30	Td	307	+	NS	1/8
31	H	311	+	–	0/8
32	F	311	+	–	0/8
33	B	311	+	–	0/8
34	Td	311	+	*	5/8

All animals’ medial retropharyngeal lymph nodes were tested for CWD through official regulatory means by ELISA, with IHC confirmation on ELISA positives, except MNPRO ID 307, which was tested by IHC only on obex and medial retropharyngeal lymph nodes. Mann–Whitney U test: NS, rate of amyloid formation is not 0 but not statistically significant from the corresponding negative controls; –, rate of amyloid formation is 0 in the given time period; ****p < 0.0001; ***p < 0.001; **p < 0.01; *p < 0.05. RT-QuIC analyses of the forelimb (F), hindlimb (H), backstrap (B), tenderloin (Td), and tongue (Tg) muscles were performed with the researcher blinded to official CWD testing results (see methods). The freeze–thaw method was used for sample processing and RT-QuIC was performed at 45 °C.

**Figure 4:**
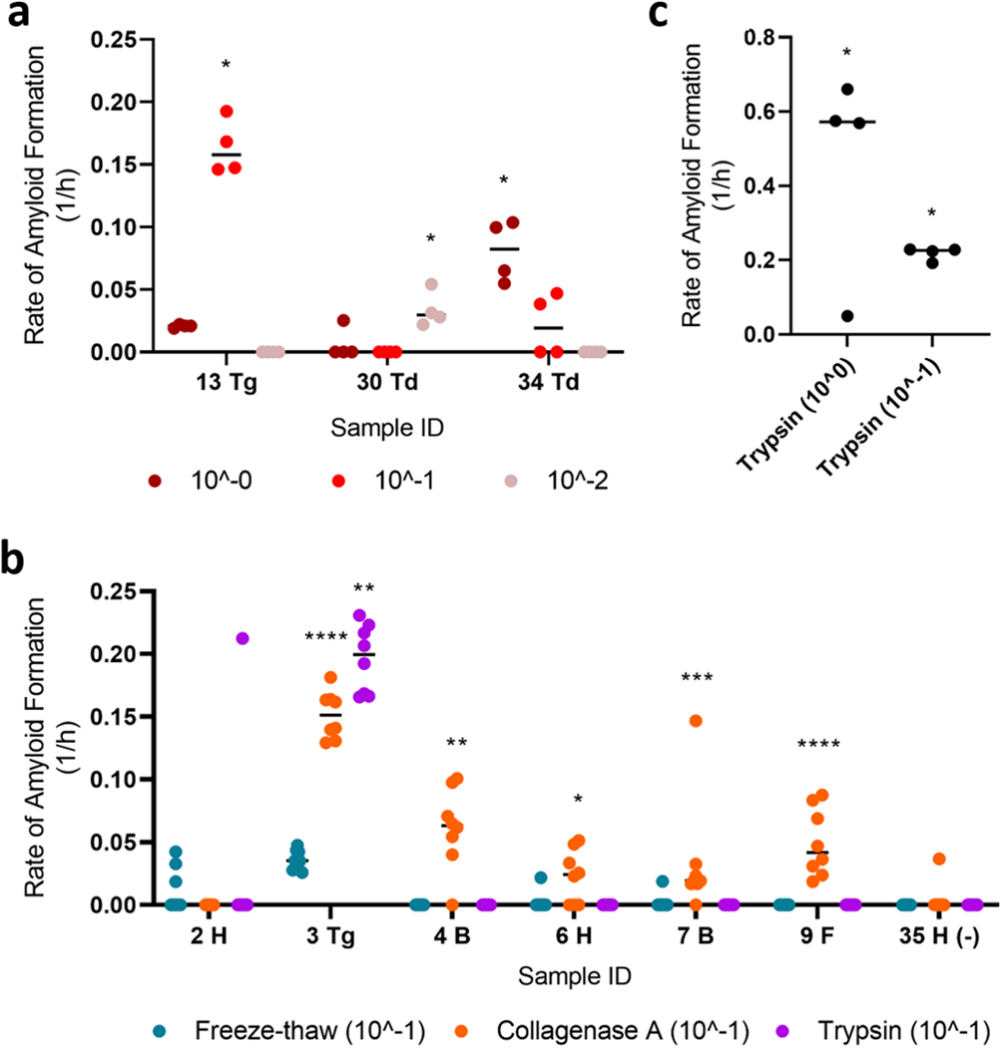
Comparison of different methods used to extract CWD prions from skeletal muscles of WTD. (**a**) Rate of amyloid formation (RAF) of samples diluted to different concentrations was visualized. (**b**) RAF from RT-QuIC was plotted for a subset of samples treated by different extraction methods, including freeze–thaw, collagenase A, and trypsin. The final suspension was diluted to 10^−1^. (**c**) RAF of trypsin-digested sample number 3 (tongue; undiluted and diluted to 10^−1^) was diagramed. Statistical result for each sample when compared with its respective negative control was indicated (****p < 0.0001; ***p < 0.001; **p < 0.01; *p < 0.05). *B* backstrap, *F* forelimb, *H* hindlimb, *Td* tenderloin, *Tg* tongue.

Notably, the samples used for this experiment were undergoing various degrees of autolysis. Hypothesizing that this may influence RT-QuIC’s ability to detect prion-seeding activity by changing the optimal dilutions of the processed homogenate, we again looked at prion-seeding activities using serial dilutions of a selected number of samples. As expected, the dilution with adequate positive wells for samples no longer consistently converged at 10^–1^ (Fig. 4a), suggesting that the freeze–thaw method is not suitable for muscle tissue samples of sub-optimal quality. To investigate whether other tissue processing methods would improve the detection of CWD prion-seeding activity in RT-QuIC, given the sub-optimal tissue preservation described above, we examined a subset of samples using enzymatic digestions (collagenase A and trypsin) instead of the freeze–thaw method. We hypothesized that collagenase A and/or trypsin would sufficiently digest potential inhibitors and/or further “release” CWD prions to a degree where extensive dilution of the processed homogenates was unnecessary. Surprisingly, collagenase A digestion still required a tenfold dilution similar to the freeze–thaw method (Supplementary Fig. 2) although it appeared to be more sensitive [i.e., identified more muscle samples as RT-QuIC positive from CWD positive animals (Fig. 4b)]; however, this was not observed when we re-tested a subset of neck muscle samples. In addition, we confirmed that the collagenase method did not appear to produce false-positive RT-QuIC signals (Supplementary Fig. 3). Alternatively, trypsin digestion produced an extremely high RAF without requiring the tenfold dilution even though its sensitivity did not improve upon the freeze–thaw method in the given sample set (Fig. 4c).

We note that all methods used in this study did result in positive prion-seeding activities using RT-QuIC on muscle tissue (Fig. 4b). The results reported here indicate that the freeze–thaw method may not be enough to facilitate RT-QuIC detection of CWD prions in aged or decomposing muscles but has utility for freshly collected samples.

## Discussion

CWD is an emerging infectious prion disease currently affecting cervid populations across three continents and negatively influencing all cervid-related industries within impacted regions. Infected animals can remain asymptomatic for months while shedding CWD prions through excreta^22,25^, thus making the identification of early-stage CWD-infected animals based on external diseased phenotypes impossible. Antibody-based ELISA and IHC tests are the current diagnostic standards for CWD. Despite their reliability, such immunodetection methods have limited sensitivity and application across various tissues and body excreta in comparison to in vitro amplification methods for prion detection, such as PMCA and RT-QuIC^9^. Of the available in vitro amplification methods, RT-QuIC is well-suited as a CWD screening tool because it can be easily scaled up as required by industrial applications. Given the continued spread of CWD, and uncertainty surrounding potential health risks to both animals and humans due to the consumption of CWD-positive venison^1^, it is clear that a highly sensitive and reliable diagnostic method to detect CWD prions in skeletal muscles of cervids is needed.

In this study, we tested methods aimed at extracting and enriching PrP^CWD^ from WTD skeletal muscles for prion detection by RT-QuIC. We first found that CWD prions were present in bulk sampled neck muscles (*brachiocephalicus*/*sternocephalicus*) of CWD positive animals (Fig. 1a)^32^. This result prompted us to investigate the general distribution of prions in skeletal muscles from the tongue, forelimb, mid-truck, and hindlimb of CWD positive WTD tissue-sets available in our biorepository. We found that in addition to the neck, PrP^CWD^ is also present in a variety of skeletal muscle tissues (described above; Table 2; Fig. 3). Our results, based on a sampling of various muscle groups, suggest that CWD prions are distributed across CWD infected WTD skeletal muscles. Additional research is needed to determine the full extent to which CWD prions occur within particular muscle tissue types of infected animals, including intra- and inter-individual variation of CWD prion accumulation in WTD muscles.

It remains to be determined whether CWD prions are detectable in skeletal muscles that were not sampled herein or in similar studies using amplification-based methods, such as PMCA and RT-QuIC. Although we were unable to detect PrP^CWD^ across all muscle types within a given CWD positive animal, this result is expected because the successful detection of PrP^CWD^ within an infected individual, and particular tissue type, can be impacted by multiple factors. With respect to the neck samples screened here, we only had access to unilaterally sampled muscles harvested from individual WTD heads and it has been shown recently that PrP^CWD^ may not be bilaterally present in select tissues^32,35^. The 10 positive animals (originating from wild WTD herds) selected for testing of neck muscles were strongly positive across multiple tissues and likely were in relatively advanced, yet pre-clinical stages of the disease (i.e., no clinical signs were observed at the time of euthanasia)^32^. The stability of prions may vary depending on strains^36^ and, to date, no RT-QuIC method is available to detect particular CWD strain differences. Further, the progression of CWD affects the deposition of prions in peripheral tissues and it is unclear at what time in the disease progression that prions accumulate in WTD muscles. We note the neck muscles used herein were frozen less than 12 h after collection; however, the other muscle tissues were at various stages of decomposition and underwent multiple freeze–thaw cycles prior to our possession. This difference in tissue preservation and quality potentially accounts for the reduced sensitivity of RT-QuIC upon application, an observation that suggests an altered balance of RT-QuIC inhibitors and active prion seeds and/or degradation of particular CWD prion strains in decaying tissue. Thus, we recommend muscles for RT-QuIC-based analyses of CWD prions be frozen (at either –20 °C or –80 °C) as soon as possible after collection, ideally less than 24 h.

Based on the results from well-preserved neck muscles, we posit that the freeze–thaw method has the most potential for large-scale diagnostic screening of venison, as it is cheaper and easier to perform. For samples with heavy prion loads, such as tongue, all methods used in this study agreed on the positivity of prion-seeding activity. For poorly preserved sample types, collagenase A outperformed the freeze–thaw method and trypsin digestion in terms of identifying more RT-QuIC positive muscle samples from CWD positive animals. Surprisingly, trypsin digestion yielded a high RAF and did not require additional dilutions of the final resuspension as needed by other methods. This could be due to the digestion of protein inhibitors by trypsin and/or superior ability of trypsin to free prions from examined tissues. Additional optimization of the methods presented here is needed for protocols focused on suboptimal sample types. It is possible that the prion seeding activity we detected in the collected muscle tissues is from non-muscle cell types as reported by Daus et al.^28^. However, the cellular origin of PrP^CWD^ in skeletal muscle, whether in myocytes, erythrocytes, neurons, epithelial cells, or any other cell type, is inconsequential to the recommendations of not consuming venison from CWD-positive animals or the potential for RT-QuIC-based venison screening as venison products are a matrix of multiple tissues and cell types.

Our findings suggest that CWD prions occur throughout an array of WTD muscles and further investigation, from an anatomical perspective, is needed to understand the extent of this distribution. Future studies focusing on larger sample sizes with systematic, bilateral samplings of well-preserved muscle samples throughout the body are needed to assess, validate, and improve the presented method for its application, as well as quantify the load of CWD prions present. Longitudinal characterization of prion deposition (i.e., using cervid challenge experiments) in a variety of high-quality muscle samples, such as those conducted for saliva, lymphoid tissues, and feces is needed to better understand the pathophysiology of CWD in deer and other cervids. Our study provides the foundation for the development of RT-QuIC-based screening of venison and venison-related products associated with food processing pipelines for CWD-prions.

## Methods

### Experimental design

The RT-QuIC muscle protocols (freeze–thaw and enzymatic digestion, detailed below) were initially used on a small subset of CWD positive and not-detected neck muscle tissue samples. After refining our methods, we then tested the protocol on a larger set of neck muscles from ten CWD positive and ten CWD not detected deer, with CWD status determined by ELISA, IHC, and RT-QuIC analyses on lymphoid tissues from the animals reported by Schwabenlander et al.^32^. To blind investigators, researcher “A” subsampled approximately 300 mg of each sample, placed them individually in 1.5 ml tubes, and re-labeled them in a randomized numerical order. Researcher “B” carried out the muscle processing and RT-QuIC and was blinded to the original identity of the samples. ELISA and IHC results for animals examined herein are presented in Supplementary Table 1. Researcher “B” was unblinded after the first pass of all samples; investigation of different extraction methods was done after unblinding.

### Sample collection

RT-QuIC protocol development was initially performed utilizing neck muscle (*brachiocephalicus*/*sternocephalicus*) tissue samples collected from wild WTD through 2019 agency culling operations in southeast Minnesota conducted by the Minnesota Department of Natural Resources in conjunction with USDA APHIS Wildlife Services as described by Schwabenlander et al.^32^ (Table 1). Muscle tissue samples for the quantitative comparison study were obtained through disposal or necropsy of farmed and wild WTD at the University of Minnesota Veterinary Diagnostic Laboratory and were stored at − 20 °C. All farmed and wild WTD examined in our study have been independently tested through official regulatory means by the National Veterinary Services Laboratories or Colorado State University, respectively, for CWD infection based on immunodetection analysis (ELISA and/or IHC) of the brain and/or lymphatic tissue for the presence of PrP^CWD^ (Table 2).

### Muscle preparation

#### Freeze–thaw method

This method was inspired by a combination of existing RT-QuIC protocols^20,25,28^. Muscles were stored at − 20 °C within 12 h after collection then transferred to − 80 °C until tested. 10% (weight/volume) muscle homogenates in 1× PBS were prepared in tubes with 1.5 mm diameter zirconium oxide beads using a Beadbug homogenizer at top speed for 180 s. The homogenates underwent three cycles of flash freeze–thaw consisting of 3 min in dry ice and 3 min at 37 °C. The homogenates were subjected to additional homogenization at top speed for 180 s using the Beadbug homogenizer. The mixtures were then centrifuged at 5000 rpm for 3 min. 500 µl of supernatants were used for centrifugation at 15,000 rpm, 4 °C for 40 min. The resultant pellets were resuspended in 100 µl of 1× PBS then incubated with 7 μl of 4% (w/v) phosphotungstic acid (Sigma-Aldrich) in 0.2 M magnesium chloride. The mixtures were then incubated at 37 °C and 1500 rpm for 1 h in a ThermoMixer (Eppendorf) before being subjected to centrifugation for 30 min at 15,000 rpm, 4 °C. Pellets were resuspended in 10 µl of 0.1% (v/v) SDS/PBS/N2. 2 μl of 10-1 diluted resuspension was used for optimal result.

#### Collagenase A and trypsin digestion method

This method for RT-QuIC was modified from the PMCA method developed by Daus et al.^28^. 10% (weight/volume, w/v) muscle homogenates and 180-s homogenization were carried out as described above. 350 μl homogenates were mixed with equal volume of 2× collagenase A [4 mM CaCl_2_ and 0.5% (w/v), Roche] or trypsin (Gibco) solutions. The mixture was incubated at 37 °C, 700 rpm for four hours. After being homogenized again for 90 s, the mixtures were centrifuged at 5000 rpm for 3 min at 4 °C. The supernatant was then transferred to another tube and mixed with an equal volume of 2× protease inhibitor cocktail (Sigma-Aldrich). This was followed by steps including centrifugation at 15,000 rpm for 40 min as the freeze–thaw method. 2 μl of the final suspensions were diluted tenfold and added to the RT-QuIC reaction. The additional dilution was not necessary for trypsin digestion.

### RT-QuIC substrate preparation and reaction conditions

Recombinant hamster PrP (HaPrP90-231; provided by NIH Rocky Mountain Laboratory) production and filtration followed the methods of Schwabenlander et al.^32^. All ingredients of RT-QuIC master mix (1× PBS, 500 µM EDTA, 50 µM ThT, 300 mM NaCl, and 0.1 mg/ml HaPrPrP) were filter-sterilized through 0.22 µm PVDF filters. 98 µL of the master mix was pipetted into wells of 96-well black clear bottoms plates. The plate was sealed with clear tape after 2 µL samples were added. Plates were then shaken on BMG FLUOstar Omega microplate readers (BMG LABTECH Inc., Cary, North Carolina, USA) at 700 rpm (57 s double orbital shaking followed by 83 s resting). Fluorescence was recorded after 21 shake/rest cycles using a 450 nm excitation filter and 480 nm emission filter. The gain was set to 1600. The machine performed 21 flashes/well and no well-scan was conducted. 45 °C, 50 °C, and 55 °C were used. 55 °C was only used for investigating whether decomposing tissues would have converging dilutions. 50 °C was used for enzymatic digestions.

### Data analysis

Statistical analysis and plotting of fluorescence data from RT-QuIC were conducted using GraphPad Prism version 9.0 for Windows, GraphPad Software, San Diego, California USA, www.graphpad.com. RT-QuIC data from four or eight replicates were used for calculating the rate of amyloid formation (RAF) for muscles, which is defined by the inverse of the time to reach the fluorescent threshold^15^. The threshold was calculated as ten standard deviations above the average baseline fluorescence unless otherwise specified. We observed variable RAF values across the four microplate readers used for our analyses (i.e. plate reader one consistently exhibited earlier amyloid seeding rates vs. plate readers two, three, and four). In no instance did this impact our positive or negative controls. However, due to RAF differences among plate readers, this threshold could not be applied to all plates. In these rare circumstances, the threshold was calculated as two times the background fluorescence in each well. The differences in RAF calculated by these two methods for a true RT-QuIC positive sample is usually less than 0.01, therefore not influencing general comparisons of RAF among plates. The one-tailed Mann–Whitney unpaired u-test was used to test the average difference between samples and corresponding negative controls on the same plates. Quantitative analysis of CWD prion load in muscles was conducted as described by Henderson et al.^15^.

### Animal research statement

No white-tailed deer were euthanized specifically for the research conducted herein and all tissues were secured from dead animals or loaned for RT-QuIC analyses. For these reasons, the research activities conducted herein are exempt from review and approval by the University of Minnesota Institutional Animal Care and Use Committee (as specified https://research.umn.edu/units/iacuc/submit-maintain-protocols/overview). All CWD positive deer were submitted to the University of Minnesota College of Veterinary Medicine for disposal of infectious prions and were sampled prior to their disposal. White-tailed deer were euthanized by the Minnesota Department of Natural Resources (MN DNR) for annual culling efforts to control the spread of CWD in Minnesota following MN DNR state regulations and euthanasia guidelines established by the Animal Care and Use Committee of the American Society of Mammalogists^37^. All methods and all experimental procedures carried out during the course of this research followed University of Minnesota guidelines and regulations as approved by the Institutional Biosafety Committee under protocol #1912-37662H. This study was also carried out in compliance with the ARRIVE guidelines (https://arriveguidelines.org). We confirm that no human tissues were used for the research performed herein.

### Field research

CWD negative animals were euthanized by the Minnesota Department of Natural Resources for routine annual culling efforts to control the spread of CWD in Minnesota and were not sampled specifically for the current study. Tissue samples were provided by the MN DNR.

## Supplementary Information

Supplementary Figures.

Supplementary Table 1.

## References

[1] Osterholm MT (2019). Chronic wasting disease in cervids: Implications for prion transmission to humans and other animal species. MBio.

[2] Prusiner SB (1982). Novel proteinaceous infectious particles cause scrapie. Science.

[3] Prusiner SB (1989). Creutzfeldt-Jakob disease and scrapie prions. Alzheimer Dis. Assoc. Disord..

[4] Ford MJ, Burton LJ, Morris RJ, Hall SM (2002). Selective expression of prion protein in peripheral tissues of the adult mouse. Neuroscience.

[5] Barria M, Libori A, Mitchell G, Head M (2018). Susceptibility of human prion protein to conversion by chronic wasting disease prions. Emerg. Infect. Dis. J..

[6] Hannaoui S, Schatzl HM, Gilch S (2017). Chronic wasting disease: Emerging prions and their potential risk. PLoS Pathog..

[7] FDA. Use of Material from Deer and Elk in Animal Feed. http://www.fda.gov/media/69936. Accessed 05 Feb 2021.

[8] FSIS, USDA. Notice 16-20. Voluntary inspection of cervid animals tested for chronic wasting disease. (2020).

[9] Haley NJ, Richt JA (2017). Evolution of diagnostic tests for chronic wasting disease, a naturally occurring prion disease of cervids. Pathogen (Basel, Switzerland).

[10] Haley NJ (2020). Management of chronic wasting disease in ranched elk: Conclusions from a longitudinal three-year study. Prion.

[11] Kramm C, Soto P, Nichols TA, Morales R (2020). Chronic wasting disease (CWD) prion detection in blood from pre-symptomatic white-tailed deer harboring PRNP polymorphic variants. Sci. Rep..

[12] Weber P (2006). Cell-free formation of misfolded prion protein with authentic prion infectivity. Proc. Natl. Acad. Sci..

[13] Wilham JM (2010). Rapid end-point quantitation of prion seeding activity with sensitivity comparable to bioassays. PLoS Pathog..

[14] Atarashi R, Sano K, Satoh K, Nishida N (2011). Real-time quaking-induced conversion: A highly sensitive assay for prion detection. Prion.

[15] Henderson DM (2015). Quantitative assessment of prion infectivity in tissues and body fluids by real-time quaking-induced conversion. J. Gen. Virol..

[16] Davenport KA, Hoover CE, Denkers ND, Mathiason CK, Hoover EA (2018). Modified protein misfolding cyclic amplification overcomes real-time quaking-induced conversion assay inhibitors in deer saliva to detect chronic wasting disease prions. J. Clin. Microbiol..

[17] Clare EH, Kristen AD, Davin MH, Mark DZ (2017). Endogenous brain lipids inhibit prion amyloid formation in vitro. J. Virol..

[18] Orrù CD, Wilham JM, Vascellari S, Hughson AG, Caughey B (2012). New generation QuIC assays for prion seeding activity. Prion.

[19] Abdel-Haq H (2015). Factors intrinsic and extrinsic to blood hamper the development of a routine blood test for human prion diseases. J. Gen. Virol..

[20] Elder AM (2015). Immediate and ongoing detection of prions in the blood of hamsters and deer following oral, nasal, or blood inoculations. J. Virol..

[21] Denkers ND, Henderson DM, Mathiason CK, Hoover EA (2016). Enhanced prion detection in biological samples by magnetic particle extraction and real-time quaking-induced conversion. J. Gen. Virol..

[22] Henderson DM (2015). Longitudinal detection of prion shedding in saliva and urine by chronic wasting disease-infected deer by real-time quaking-induced conversion. J. Virol..

[23] Hoover CE (2017). Pathways of prion spread during early chronic wasting disease in deer. J. Virol..

[24] Cooper SK (2019). Detection of CWD in cervids by RT-QuIC assay of third eyelids. PLoS One.

[25] Tennant JM (2020). Shedding and stability of CWD prion seeding activity in cervid feces. PLoS One.

[26] Otero A (2019). Prion protein polymorphisms associated with reduced CWD susceptibility limit peripheral PrP(CWD) deposition in orally infected white-tailed deer. BMC Vet. Res..

[27] Jewell JE, Brown J, Kreeger T, Williams ES (2006). Prion protein in cardiac muscle of elk (*Cervus elaphus nelsoni*) and white-tailed deer (*Odocoileus virginianus*) infected with chronic wasting disease. J. Gen. Virol..

[28] Daus ML (2011). Presence and seeding activity of pathological prion protein (PrPTSE) in skeletal muscles of white-tailed deer infected with chronic wasting disease. PLoS One.

[29] Angers RC (2006). Prions in skeletal muscles of deer with chronic wasting disease. Science.

[30] Olszowy KM (2014). Six-year follow-up of a point-source exposure to CWD contaminated venison in an Upstate New York community: Risk behaviours and health outcomes 2005–2011. Public Health.

[31] Haley NJ (2016). Antemortem detection of chronic wasting disease prions in nasal brush collections and rectal biopsy specimens from white-tailed deer by real-time quaking-induced conversion. J. Clin. Microbiol..

[32] Schwabenlander MD (2021). Comparison of chronic wasting disease detection methods and procedures: Implications for free-ranging white-tailed deer (*Odocoileus Virginianus*) surveillance and management. J. Wildl. Dis..

[33] Wisconsin Department of Natural Resources. Wisconsin Department of Natural Resources. https://dnr.wisconsin.gov/. Accessed 05 Feb 2021.

[34] Bosque PJ (2002). Prions in skeletal muscle. Proc. Natl. Acad. Sci..

[35] Bloodgood J, Kiupel M, Melotti J, Straka K (2021). Chronic wasting disease diagnostic discrepancies: The importance of testing both medial retropharyngeal lymph nodes. J. Wildl. Dis..

[36] Angers RC (2010). Prion strain mutation determined by prion protein conformational compatibility and primary structure. Science.

[37] Sikes RS, The Animal Care and Use Committee of the American Society of Mammalogists (2016). 2016 Guidelines of the American Society of Mammalogists for the use of wild mammals in research and education. J. Mammal..

