# Supplementary material for: RT-QuIC detection of CWD prion seeding activity in white-tailed deer muscle tissues
Source: Sci Rep. 2021 Aug 18;11:16759. doi: 10.1038/s41598-021-96127-8 (PMC8373970; doi:10.1038/s41598-021-96127-8)
Supplement: Supplementary file 1 — Supplementary Figures. [file 41598_2021_96127_MOESM1_ESM.docx]

**Supplementary Fig. 1**

**
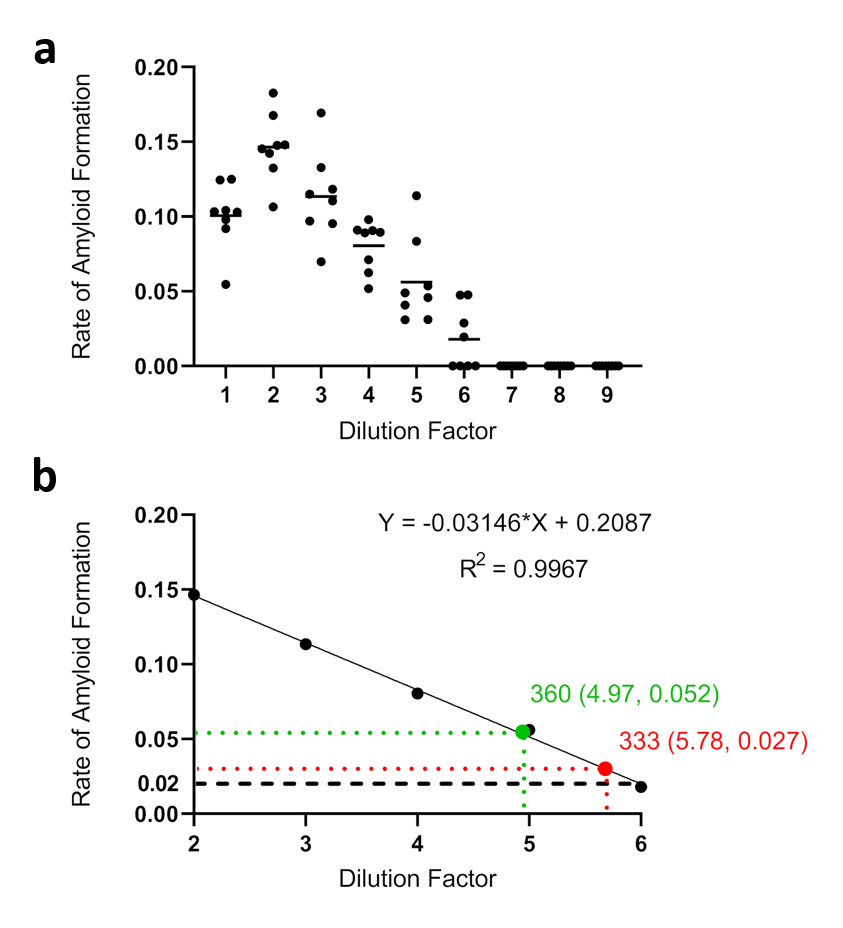
**

Comparison of RAF (rate of amyloid formation) between lymphoid and skeletal muscle tissues. **a.** RAF of 10% lymphoid tissue homogenates was plotted against dilution factors ranging from 1 to 9. The RT-QuIC experiment was set at 45°C. 8 replicates were conducted for all dilutions. **b.** The average of RAF from dilutions in A) with dilution factors ranging from 2-6 was fitted on a linear model. Equation and R^2 values are indicated. RAF for neck muscle samples from animal 333 and 360 processed using the freeze-thaw method and normalized to the lymphoid tissue used in a) were plotted.

Result: Prion load using muscles processed by the freeze-thaw method based on RAF from RT-QuIC results is equivalent to 10^-6^~10^-7^ (2/1000 * 10/2 * 10 * 10^-5^~10^-6^) that of lymphoid tissues, the RAF of which is 10 times lower than the brain. Therefore, in our study, the lymphoid/muscle ratio is 10^6^~10^7^ and the calculated brain/muscle ratio is 10^5^~10^6^. This is 100 to 1000 times lower than the brain/muscle ratio of 10^3^ reported by Bosque et al.

**Supplementary Fig. 2**

**
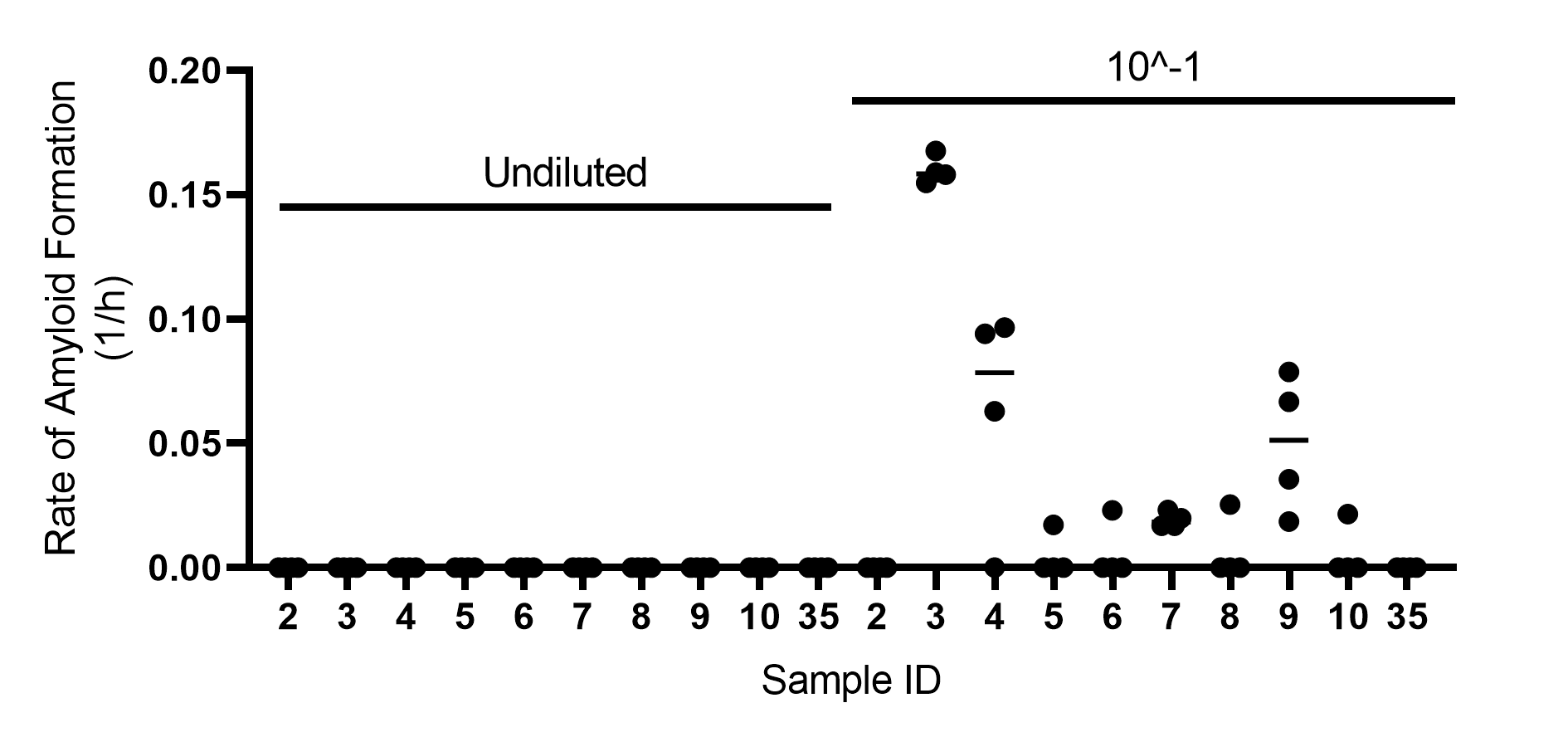
**

Collagenase A-processed samples need to be diluted to facilitate prion-seeding activity detection in RT-QuIC. A subset of samples with undiluted final suspension and that diluted to 10^-1 were tested on the same plate.

**Supplementary Fig. 3**


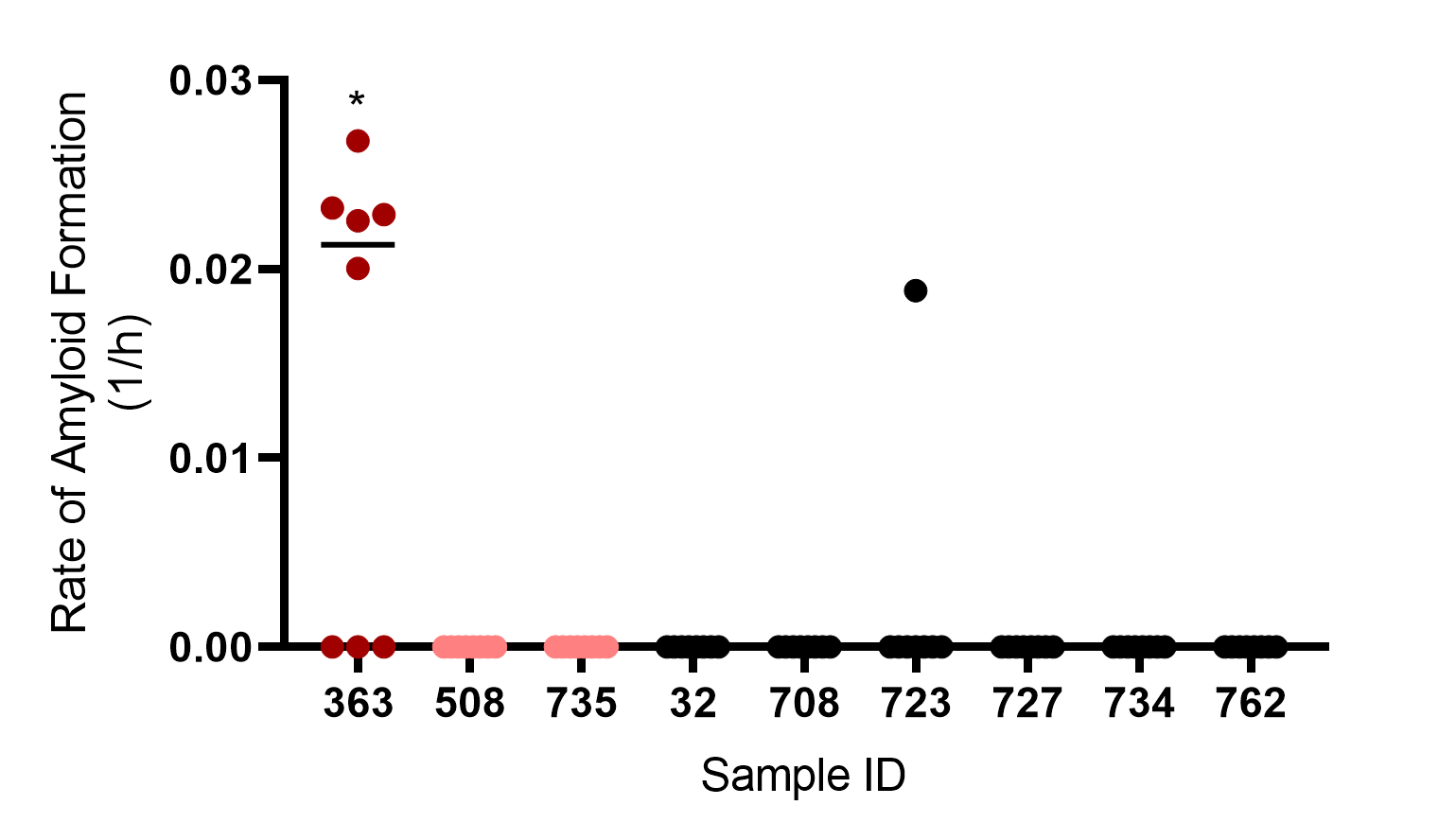


Collagenase A processing does not produce significant RT-QuIC false positives for neck muscles.
